# Deep Learning for LiDAR Point Cloud Classification in Remote Sensing

**DOI:** 10.3390/s22207868

**Published:** 2022-10-16

**Authors:** Ahmed Diab, Rasha Kashef, Ahmed Shaker

**Affiliations:** 1 Department of Civil Engineering, Toronto Metropolitan University, Toronto, ON M5B 2K3, Canada; 2 Electrical, Computer, and Biomedical Engineering, Toronto Metropolitan University, Toronto, ON M5B 2K3, Canada

**Keywords:** point clouds, deep learning, remote sensing

## Abstract

Point clouds are one of the most widely used data formats produced by depth sensors. There is a lot of research into feature extraction from unordered and irregular point cloud data. Deep learning in computer vision achieves great performance for data classification and segmentation of 3D data points as point clouds. Various research has been conducted on point clouds and remote sensing tasks using deep learning (DL) methods. However, there is a research gap in providing a road map of existing work, including limitations and challenges. This paper focuses on introducing the state-of-the-art DL models, categorized by the structure of the data they consume. The models’ performance is collected, and results are provided for benchmarking on the most used datasets. Additionally, we summarize the current benchmark 3D datasets publicly available for DL training and testing. In our comparative study, we can conclude that convolutional neural networks (CNNs) achieve the best performance in various remote-sensing applications while being light-weighted models, namely Dynamic Graph CNN (DGCNN) and ConvPoint.

## 1. Introduction

The light detection and ranging (LiDAR) mapping generate precise spatial information about the shape and surface components of the Earth. Advancements in LiDAR mapping systems and their technologies have been proven to examine natural and manmade environments across various scales with higher accuracy, precision, and flexibility [1]. LiDAR Remote sensing provides an accurate 3D representation of scanned areas with many features that provide great performance for various applications. Such applications include Digital Elevation Model (DEM), Digital Surface Model (DSM), and Digital Terrain Model (DTM) generation, which, combined with intensity data, achieve excellent performance in urban land cover classification [2]. Some other urban applications include pavement crack detection [3], collapsed building detection [4], road markings and fixtures extraction and classification [5], cultural heritage classification [6], and change detection [7]. Because LiDAR is sensitive to variations in vertical vegetation structure, it makes it very effective for natural resources [8] and forest applications [7], such as tree species classification [9]. Additionally, full-waveform LiDAR adds more advantages to using LiDAR in forestry applications [10].

Various deep learning models have been developed with outstanding performance for data classification on point cloud datasets in multiple applications. Existing deep learning methods for point cloud classifications involve architectures based on the traditional neural network, the Multi-Layer Perceptron (MLP). These models are called PointNet-Based as they build on the pioneering work of PointNet [11]. PointNet is a great performer that is very lightweight but suffers from local information loss. Global features are features of a scene, object, or image that describe it as a whole, compared to local features that are extracted at different points and represent patches of the scene or image [12]. PointNet++ [13] mitigates the loss by building a feature aggregation pyramid to learn hierarchically, similar to how a traditional Convolutional network learns. One of the biggest challenges of using LiDAR point clouds in deep learning is the unstructured shapes of the point cloud data; a convolutional kernel that works on uniform grid-structured data cannot be directly applied to the raw point cloud. A convolutional neural network can better capture spatial features, which performs better than a traditional neural network while being more lightweight than most handcrafted models. The convolutional neural network is structured as a convolution layer, non-linearity, e.g., Rectified linear unit (ReLU), and pooling layers to distil features from low-level to high-level [14]. Applying CNNs on point clouds involves the 2D projection of the point cloud to obtain images that can then be fed into traditional convolution layers in a convolutional neural network. Another approach is resampling or restructuring the point cloud into uniform volumetric grids using occupancy functions and 3D convolutional layers to create the CNN or to design novel convolutional layers that can operate on pointsets and the custom convolution operation to build the CNN.

This paper provides a roadmap for current DL deep learning models for LiDAR point cloud classifications in remote sensing. Existing deep learning methods can be classified as projection-based and point-based models. Each category enjoys specific characteristics; however, they show some limitations. Thus, this paper summarizes the significant subcategories: 2D projection, Multiview projection, voxelization, Convolutional-based networks, and graph convolutional networks. Additionally, we cover some examples that encompass most of the fundamentals within each subcategory. Remote sensing applications require different datasets or workflows; thus, we cover some examples from remote sensing that employ or build upon computer vision models. Our comparative analysis shows that DGCNN and ConvPoint have shown the best performance in various remote-sensing applications while being light-weighted models. The rest of this paper can be organized as Section 2 focuses on LiDAR point cloud data and processing overview, Section 3 introduces the primary computer vision deep learning models that are often used to classify 3D data, and Section 4 presents Point cloud computing tasks that are common in remote sensing applications, Section 5 introduces the benchmark 3D datasets used in training and testing of deep learning models grouped as objects, indoor, arial scanned, mobile scanned, and terrestrial scanned datasets, Section 6 shows the evaluation metrics commonly used to measure and benchmark model performance; Section 7 provides a comparative analysis of existing models on different datasets for different classification tasks. Finally, Section 8 concludes the paper.

## 2. LiDAR Point Clouds

**Figure 1:**
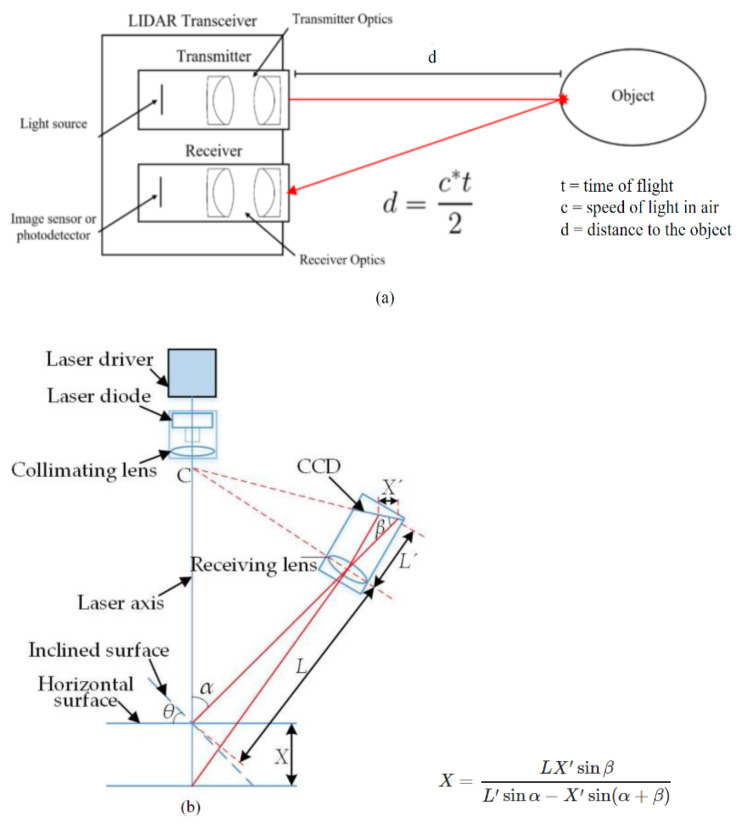
Time of Flight LiDAR sensor calculation (**a**) [16] and triangulation-based LiDAR calculation (**b**) [17].

A typical LiDAR system in remote sensing uses a laser, Global Positioning System (GPS) and an Inertial Measurement Unit (IMU) to approximate the heights of objects on the ground. Discrete LiDAR data are generated; each point represents high energy points along with rebounded energy. Discrete LiDAR points contain each point’s x, y, and z values. The z value is used to obtain height. The LiDAR data can estimate surface structures with various methods [15]. The raw LiDAR data are delivered as points, known as point clouds, that can be further processed to create Digital Elevation Models (DEMs) or Triangulated Irregular Networks (TINs) [1]. Point data are commonly stored in LAS (LASer) format, regarded as an industry standard that contains information in a binary file specific to the LiDAR nature of data without being complex [15]. The LiDAR data can also contain other information such as the intensity of the rebounds, the point classification (if applicable), number of returns, time, and source of each point [1,15]. LiDAR scanners use a laser pulse to measure the distance from the sensor using the time for the laser pulse to return in the case of time-of-flight sensors (Figure 1a) [16] or using the triangulation angle on the optical sensor for triangulation-based scanners (Figure 1b) [17]. The LiDAR scanners then generate an [x, y, z] position relative to the sensor’s locations based on the distance from the sensor and the degrees of rotation of the sensor, such as pitch, roll, and yaw [18]. Most LiDAR sensors also measure the intensity of the return signal, which can be used to differentiate between different surface types with different reflectivity [1]. Additionally, the sensor is often paired with a GPS and an IMU to capture data required for georeferencing and mapping of the point cloud.

For supervised classification, a significant challenge when working on LiDAR point clouds is the variation in density inherent in the nature of the data. The density of similar objects is also varied, as it depends on the speed of the vehicle mounting the sensor. Some areas will be too dense and expensive to process, requiring some form of downsampling. Other regions of a point cloud will have few or no points present. Additionally, for LiDAR point clouds that include intensity values, the intensity of the same object could be affected by different conditions and result in the same object having slightly different intensities [18].

## 3. Point Cloud Computing

Remote sensing data go through multiple processing steps to generate information that can be consumed for production. Over the past few years, deep learning has been applied to almost all remote sensing data processing aspects. Most notably, classification and segmentation tasks. Regarding remote sensing 3D LiDAR point clouds, there is limited interest in whole scene classification and more in semantic classification or segmentation tasks. Some other examples of deep learning tasks tackled by deep learning include change detection, registration, fusion, and completion.

Traditionally, deep learning classification describes classifying an entire scene or an object as belonging to a specific class as a whole. One example of classification tasks that use 3D point clouds in remote sensing is the classification of tree species or roof types previously segmented. However, remote sensing classification tasks involve semantic classification and segmentation rather than aiming to identify an entire scene or object to a single class. A significant example of semantic classification is Land use/Land cover classification of Terrestrial and Arial Laser scanned (TLS/ALS) data. Segmentation divides and assigns the data into different target classes and is split into three types, semantic, instance, and panoptic segmentation [19]. Semantic segmentation assigns every point/pixel from the input data to one of the target classes without distinguishing different objects; for example, all tree points will be labelled trees. Instance segmentation involves identifying and labelling objects belonging to target classes while distinguishing them from each other, such as tree1, tree2, etc. Panoptic segmentation classifies every point/pixel in the input as part of a class while distinguishing separate objects of a class from each other [19].

The most common application of image fusion in LiDAR remote sensing is the fusion of 3D point clouds and RGB images to train a deep learning model for classification and segmentation tasks [20,21,22]. The features extracted from both types of data are used to enhance the performance of each class in the application of each class. Registration is the process of matching and aligning two or more images or point clouds in the case of LiDAR data obtained from different viewpoints and/or using different sensors; one example is illustrated in [23], which achieves state-of-the-art performance. Completion is the process of filling in missing information from datasets that could result from the limitations of the sensors, conditions at the time of data capture, or the method of capture. For far-away distances, the spatial resolution of a LiDAR sensor is lower, sometimes resulting in finer details, such as road markings, signs, poles, etc., showing up incomplete. One example of completion can be found in [5]. Most completion tasks on LiDAR point clouds are done before training a classification model to improve performance and robustness.

## 4. Deep Learning Models

Advances have been made to produce DL models that are lightweight and efficient. Feature learning models on 3D point clouds can be categorized as projection-based and point-based models. This section briefly discusses models used as backbones or improved for newer networks.

### 4.1. Projection-Based Methods

Some projection-based models create 2D projections from 3D point clouds and use traditional 2D feature learning. This process primarily depends on projection direction (X, Y or Z—default: Z) and other aspects such as the grid (size, scale, shape). Other projection models create volumetric grids or voxels through 3D feature extraction layers.

- 2D Convolutional Neural Networks

U-Net [24]: builds on a fully convolutional model and extends it to work with few training data while providing better performance. The U-Net architecture consists of repeated two unpadded 3 × 3 convolutions followed by ReLU and downsampling 2 × 2 max pooling with stride 2. For each convolution step, the number of feature channels is doubled. In the deconvolution steps, the features are upsampled and followed by a 2 × 2 convolution that halves the number of channels. The resulting feature map goes through cropping and two 3 × 3 convolutions followed by a ReLU. The cropping is necessary because of the border pixels lost after every convolution. Finally, a 1 × 1 convolution is applied to label pixels and generate segmentation results.

DeepLab [25]: employs atrous convolution [25,26] to change the scope of convolution and extract global features while also allowing larger networks without extra parameters. DeepLab proposes Atrous Spatial Pyramid Pooling (ASPP) to segment at different scales by applying the same filters at different sampling rates and field-of-views, then the outputs are added together. To overcome the toll downsampling and max pooling operations in deep convolutional neural networks (DCNNs), DeepLab implements the fully connected Conditional Random Field (CRF) from [27], which is trained separately from the rest of the network. Iterations DeepLabV3 [28] and DeepLabV3+ [29] improve the performance of DeepLab. Unlike [25], DeepLabV3 [28] performs batch normalization within ASPP. Additionally, global average pooling is applied to the last feature map. The resulting image-level features are fed into a 1 × 1 convolution with 256 filters, then multiplied to the desired spatial dimension. DeepLabV3 abandons the CRF and replaces it with concatenating and aggregating the resulting features and passing them through another 1 × 1 convolution with 256 filters before computing the final logits. DeepLabV3+ [29] uses a decoder module to refine segmentation results, especially around object boundaries. Depth-wise separable convolutions are applied to ASPP pooling and decoder modules resulting in a faster and more robust network.

VGGNet [30] evaluates the effect of increasing the network depth of a convolutional network using very small 3 × 3 convolution filters. It improves the classification performance compared to previous state-of-the-art models by pushing the depth to 16–19 weight layers. ResNet [31] adopts residual learning to every stacked layer in the convolutional network. The shortcut connections are added without increasing parameter or computation complexity. The residual learning allows deep networks with performance gain over shallower networks.

- Multiview representation

MVCNN [32] tackles 3D feature learning using traditional image-focused networks by making 2D renders of the 3D object from different angles and passing it through a standard CNN. MVCNN generates 80 views of the 3D object by placing 20 virtual “cameras” pointed at the object’s centroid, then generates 4 renders per camera at 0-, 90-, 180-, and 270-degree rotation along the axis through the camera and object center. After each image is passed through the first CNN, the outputs are aggregated at a view-pooling layer which performs element-wise maximum operation across the different input views before passing through the remaining section of the network, i.e., the second CNN.

- Volumetric grid representation

VoxNet [33] uses occupancy grids to efficiently estimate occupied, free, and unknown space provided by ranging measurements. Small (32 × 32 × 32 voxels) dense voxels are used to optimize GPU usage. VoxNet uses a more basic 3D CNN to extract and learn features, consisting of 5 of two convolution layers, a convolution and pooling layer, and two fully connected layers. The model can perform object classification in real-time while achieving state-of-the-art performance. VoxelNet [34] introduces a multi-layer voxel feature encoding (VFE) that enables inter-point interaction within a voxel. The point cloud is divided into equally spaced voxels encoded using the stacked VFE layers, allowing complex local 3D information learning. VoxelNet works on object detection using a Region Proposal Network (RPN) at the final stage to create bounding boxes.

### 4.2. Point-Based Methods

Point-based methods consume unstructured and unordered point clouds. Some of the models covered in this section are used as backbones or parts of a larger architecture, while others are adapted for remote sensing tasks with minimal modifications.

- PointNets

PointNet [11] directly consumes point cloud data for feature extraction. The network provides a unified approach to 3D recognition that can be applied for various tasks such as object classification, instance segmentation, and semantic segmentation. PointNet uses Multi-Layer Perceptrons (MLPs) combined with a joint alignment network. To hold invariance under geometric transformations, the input is passed through a T-Net module [11], where it is multiplied by an affine transformation matrix. PointNet provides great performance while remaining lightweight and computationally efficient. PointNet cannot produce local features of neighbouring points; PointNet++ [13] introduces a class pyramid feature aggregation scheme. The scheme comprises three stacked layers: the sampling layer, the grouping layer, and the PointNet layer. This allows PointNet++ to extract features in a hierarchical fashion similar to traditional image learning, reducing local information loss. PointASNL [35] is an end-to-end network that effectively deals with noisy point clouds. The two primary components of the model are the adaptive sampling (AS) and the local-nonlocal (L-NL) modules. Initially, the AS module reweighs neighbour points surrounding the initial sampled points from the farthest point sampling and then adaptively adjusts the sampled points beyond the point cloud. The L-NL module captures the neighbour and long-range dependencies of the sampled point. Self-Organizing Network (SO-Net) [36] generates a Self-Organizing Map (SOM) to simulate point cloud spatial distribution. The SOM retrieves hierarchical features from individual points and SOM nodes. A Point-to-node search is performed on the output of the SOM for each point. Each point is normalized, and features are learned through a series of fully connected layers. Node feature extraction is done through channel-wise max-pooling the point features. Final learned features are extracted using a batch of fully connected layers referred to as a small PointNet.

- (Graph) Convolutional Point Networks

ConvPoint [37] proposes continuous convolution kernels to allow arbitrary point cloud sizes. Points {q} are selected iteratively from the input point cloud {p} until the target number of points is reached through a score-based process. Using a kd-tree built on the input point cloud, K-nearest neighbour search from {p} is performed on points in {q}. A convolution operation is performed for each subset, generating the output features. Operations detailed by ConvPoint are successfully adapted for classification, part segmentation, and semantic segmentation tasks. ConvPoint can produce significant performance with time- and cost-efficient. Dynamic Graph CNN (DGCNN) [38] generates local neighbourhood graphs and applies convolution on the edges connecting neighbour point pairs. Unlike traditional graph CNNs, DGCNN uses a dynamic graph where the set of k-nearest neighbours for a point change between layers in the network and is calculated from the sequence of embeddings. The EdgeConv block introduced by DGCNN computes edge features for each input point and applies an MLP followed by channel-wise symmetric aggregation. Taylor Gaussian mixture model (GMM) network (TGNet) [39] is composed of units named TGConv that perform convolution operations parametrized by a family of filters on irregular point sets. The filters are products of geometric features expressed by Gaussian weighted Taylor kernels and local point features extracted from local coordinates. TGConv features are aggregated using parametric pooling to generate feature vectors for each point. TGNet uses a CRF at the output layer to improve segmentation results.

## 5. Benchmark Datasets

Advancements in Deep learning on point clouds have attracted more and more attention, especially in the last few years. Several publicly available datasets were also released, which helped further support research on DL development. An increasing number of methods have been introduced to deal with various challenges related to point cloud processing, including 3D shape classification, 3D object detection and tracking, 3D point cloud segmentation, 3D point cloud registration, 6-DOF pose estimation, and 3D reconstruction [18]. Table 1 briefly overviews some of the most commonly used publicly available point cloud datasets. Outdoor datasets are classified based on acquisition technique, Aerial, Mobile, or Terrestrial Laser scanned data or ALS, MLS, and TLS, respectively. The remaining datasets in this paper are indoor laser-scanned datasets and datasets of object scans. While ModelNet40 and S3DIS are not LiDAR scanned datasets, they are included as we found that they are the most commonly tested datasets for their respective tasks in remote sensing classifications. ModelNet40 dataset consists of CAD files; most point cloud network testing uses a point cloud sampled from the 3D object files. The models that used the ModelNet40 dataset outlined later in the paper are tested on the dataset by sampling the objects into a point cloud and then applying the model. Similarly, S3DIS, while not LiDAR data, is a point cloud and the models tested on it are suitable for point clouds obtained from LiDAR scans.

## 6. Performance Metrics

Various evaluation metrics have been used for segmentation, detection, and classification. The summary of the evaluation metrics [53] is shown in Table 2. Metrics for segmentation, detection, and classification are the intersection over union (IoU), mean IoU, and overall accuracy (OA) [53]. Detection and classification results are mainly analyzed using precision, recall and F1-score, which takes the true positives (TP), false positives (FP), and false negatives (FN) for calculation.

## 7. Comparative Analysis

The datasets ModelNet40, S3DIS, and Toronto3D provide an overview of benchmarks used for different classification tasks: object classification, indoor scene classification, and urban outdoor classification. Table 3 shows the performance comparison for the current 3D object classification, indoor scene segmentation, and outdoor urban semantic segmentation models using various evaluation metrics. The best-performing configuration for each model was selected. For example, using a higher sampled point cloud in ModelNet40 tests can produce better performance. Therefore, if the authors tested the models using different point counts, the best set of results is used. The results outlined in the table are obtained from the testing by each model’s respective author(s) except for the ConvPoint results on Toronto3D, which we tested for this paper. From Table 3, we can see that DGCNN and ConvPoint achieve the best performance on most datasets while being lightweight relative to models with similar performance. Additionally, these two models have been tested on multiple different tasks and different types of datasets. The major limitation of ConvPoint is that the convolutional layer introduced is a scale agnostic, i.e., the object’s size is important for scans and provides valuable information. DGCNN could be further improved by adjusting the implementation details to improve the computational efficiency of the model.

Most remote sensing papers use one of the previously outlined computer vision models. The model is deployed directly for the application dataset or modified and attached to post and/or preprocess pipelines. To further test the performance of the ConvPoint model in this paper, we have also experimentally trained ConvPoint on Toronto3D using labels such as L001, L003, and L004 and used L002 for testing. The training was run using batch size 8, block size 8, and #of points 8192 for 100 Epochs. The testing results are marked with a (*) in Table 4. Table 4 includes some applications categorized according to their dataset, performance, and remote sensing deployment. We can conclude that both DGCNN and ConvPoint have shown promising results across the different applications in remote sensing.

## 8. Conclusions and Future Directions

Recent work on the advances of deep learning on LiDAR 3D point cloud processing was analyzed and summarized. An overview of the different model types and the state-of-the-art and/or fundamental models of each type was provided. Additionally, the performance of the models was provided on datasets for different classification tasks. The strongest performing models were trending towards 3D Graph CNNs and 3D CNNs [69,70] that work directly on the raw point cloud data. These models can provide state-of-the-art performance and remain computationally lightweight. Finally, different applications of remote sensing that deploy deep learning models were overviewed. One major challenge when comparing the remote sensing models was the lack of standardized test datasets and the frequent use of proprietary datasets. Notable test datasets available are Toronto3D, Paris-Lille 3D, ISPRS 3D, and S3dIS. Future Directions would involve expanding the application of the state-of-the-art methods in autonomous driving [71,72].

## Figures and Tables

**Table 1:** Benchmark datasets for training and testing deep learning on 3D point clouds.

Dataset	Data Type	Data Format	Points/Objects	No. of Classes	Density
ModelNet40 [40]	3D CAD	OFF Files	127,915 Models	40	N/A
ISPRS 3D Vaihingen [41]	ALS LiDAR	x, y, z, reflectance, return count	780.9 K pts	9	4–8 pts/m^2^
Hessigheim 3D [42]	ALS LiDAR	x, y, z, intensity, return count	59.4 M training pts, 14.5 M validation pts	11	800 pts/m^2^
2019 IEEE GRSS Data fusion contest [43]	ALS LiDAR	x, y, z, intensity, return count	83.7 M training pts, 83.7 M validation pts	6	Very dense
AHN(3) [44]	ALS LiDAR	x, y, z, intensity, return count, additional normalization, and location data	190.3 M pts	5	20 pts/m^2^
RoofN3D [45]	ALS LiDAR	multipoints, multipolygons	118.1 K roofs	3	4.72 pts/m^2^
semanticKITTI [46]	MLS LiDAR	x, y, z, reflectance, GPS data	4.549 K pts	25 (28)	Sparse
S3DIS [47]	Indoor Structured-light 3D scanner	x, y, z, r, g, b	215.0 M pts	12	35,800 pts/m^2^
Paris-Lille-3D [48]	MLS LiDAR	x, y, z, reflectance, additional position data	143.1 M pts	10 coarse (50 total)	1000–2000 pts/m^2^
Toronto3D [49]	MLS LiDAR	x, y, z, r, g, b, intensity, additional position data	78.3 M pts	8	1000 pts/m^2^
ArCH [50]	TLS LiDAR, TLS+ALS LiDAR	x, y, z, r, g, b, normalized coordinates	102.1 M training pts, 34.0 M testing pts	6–9 depending on the scene	subsampled differently depending on the scene
Semantic3D [51]	TLS LiDAR	x, y, z, intensity, r, g, b	4.0 B pts	8	Very dense
3D Forest [52]	TLS LiDAR	x, y, z, intensity	467.2 K pts	4	15–40 pts/m^2^

**Table 2:** Performance Evaluation Metrics.

Metric	Formula	
IoU	$IoU_{i}=\frac{c_{ii}}{c_{ii}+\sum_{j\neq i}c_{ij}+\sum_{k\neq i}c_{ki}\;}$	Where *cij* is ground truth class, *i* predicted as *j*
mIoU	$mIoU=\frac{\sum_{i=1}^{N}IoU_{i}}{N}$	Where *N* is the number of classes
OA	$OA=\frac{\sum_{i=1}^{N}c_{ii}}{\sum_{j=1}^{N}\sum_{k=1}^{N}c_{jk}\;}$	
Precision	$Precision=\frac{TP}{TP+FP}$	
Recall	$Recall=\frac{TP}{TP+FN}$	
F_1_ score	$F_{1}=\frac{2TP}{2TP+FP+FN}$	
Average precision (AP)	$AP=\frac{1}{11}\underset{r\in \left\{0,1,\ldots ,1\right\}}{\sum }max_{ř:ř\geq r}p\left(ř\right)$	
Kappa coefficient	$K=\frac{N\sum_{i=1}^{k}x_{ii}-\sum_{i=1}^{k}\left(x_{i+}\times x_{+i}\right)}{N^{2}-\sum_{i=1}^{k}\left(x_{i+}\times x_{+i}\right)}$	

**Table 3:** Comparative Analysis of Deployed Models.

**ModelNet40** Object Classification										
**Method**	**OA**				**Class Average Accuracy**					
PointNet [11]	89.2				86.2					
PointNet++ [13]	91.9				-					
ConvPoint [37]	92.5				89.6					
DGCNN [38]	93.5				90.7					
MVCNN [32]	90.1				79.5					
FKAConv [54]	92.5				89.5					
VoxNet [33]	83.0				-					
SO-Net [36]	93.4				90.8					
PointASNL [35]	93.2				-					
**S3DIS** Indoor Semantic segmentation										
**Method**	**OA**				**mIOU**					
PointNet [11]	78.62				47.71					
ConvPoint/Fusion [37]	85.2/88.8				62.6/68.2					
DGCNN [38]	84.1				56.1					
PointASNL [35]	-				68.7					
TGNet [39]	88.5				57.8					
FKAConv [54]	-				68.4					
**Toronto3D** Urban MLS Semantic segmentation										
**Method**	**OA**	**mIoU**	**Road**	**Road mrk.**	**Natural**	**Bldg**	**Util. line**	**Pole**	**Car**	**Fence**
PointNet++ [13]	84.88	41.81	89.27	0.00	69.00	54.10	43.70	23.30	52.00	3.00
DGCNN [38]	94.24	61.79	93.88	0.00	91.25	80.39	62.40	62.32	88.26	15.81
TGNet [39]	94.08	61.34	93.54	0.00	90.83	81.57	65.26	62.98	88.73	7.85
MSAAN [55]	95.90	75.00	96.10	59.90	94.40	85.40	85.80	77.00	83.70	17.70
ConvPoint * [37]	96.07	74.82	97.07	54.83	93.55	90.60	82.9	76.19	92.93	12.42
[56]	93.6	70.8	92.2	53.8	92.8	86.0	72.2	72.5	75.7	21.2

**Table 4:** Overview of some deep learning contributions focused on remote sensing data.

Paper	Category	Architecture(s) Based on/Proposed	Test Dataset	Performance ^1^	Application
[5]	2D Projection	CNN, cGAN	TUM MLS 2016	85.04 *	Road marking extraction, classification, and completion
[57]	2D Projection	1D CNN, 2D CNN, LSTM DNN	ISPRS 3D Vaihingen	79.4 *	ALS Point cloud classification
[56]	2D projection Point CNN	3D Convolution U-Net	Toronto3D	70.8 ^	MLS Point cloud semantic segmentation
[58]	Multi-view Projection	MVCNN	RoofN3D	99 * Saddleback 96 * Two-sided Hip 83 * Pyramid	Roof Classification
[59]	Voxelization	Clustering, Voxelization, 3D CNN	ISPRS 3D Vaihingen	79.60 *	ALS Point cloud classification
[60]	Voxelization, 2D projection	DenseNet201	ISPRS 3D Vaihingen	83.62 *	ALS Point cloud classification
[61]	PointNet/MLP/FCL	PointNet++, Joint Manifold Learning, Global Graph-based	ISPRS 3D Vaihingen AHN3	66.2 * 83.7 *	ALS Point cloud classification
[62]	PointNet/MLP/FCL	PointNet++	Proprietary	95.4 ~	TLS Forest Point cloud Semantic Segmentation
[21]	PointNet/MLP/FCL	MSSCN, MLP, Spatial Aggregation Network	S3DIS ScanNet	89.8 ~ 86.3 ~	Point Cloud Semantic Segmentation
[55]	PointNet/MLP/FCL	MSAAN, RandLA-Net	CSPC (scene-2, scene-5) Toronto3D	64.5 ^, 61.8 ^, 75.0 ^	Point Cloud Semantic Segmentation
[63]	PointNet/MLP/FCL	PointNet T-Nets, FWNet, 1D CNN	ZORZI et al. 2019	76 *	Full-Waveform LiDAR Semantic Segmentation
[64]	Point CNN	Dconv, CNN, U-Net	ISPRS 3D Vaihingen	70.7 *	ALS Point cloud classification
[65]	Point CNN	ConvPoint, CNN	Saint-Jean NB (provincial website) Montreal QC (CMM)	96.6 ^ 69.9 ^	ALS Point cloud classification
[66]	Voxelization 3D CNN	3D CNN, DQN	ISPRS 3D Vaihingen	98.0 ~	Point cloud classification and reconstruction
[67]	Graph/Point CNN	Graph attention CNN	ISPRS 3D Vaihingen	71.5 *	ALS Point cloud classification
[68]	Graph/Point CNN	DGCNN	AHN3	89.7 *	ALS Point cloud classification
[6]	Graph/Point CNN	DGCNN	ArCH	81.4 *	Cultural Heritage point cloud segmentation

^1^ f1-Score is denoted by *, mIOU is denoted by ^ and OA is denoted by ~.

## Data Availability

Not applicable.
